# Supplementary material for: Staphylococcus aureus lipoproteins promote abscess formation in mice, shielding bacteria from immune killing
Source: Commun Biol. 2021 Mar 30;4:432. doi: 10.1038/s42003-021-01947-z (PMC8010101; doi:10.1038/s42003-021-01947-z)
Supplement: Supplementary file 2 — Supplementary Information [file 42003_2021_1947_MOESM2_ESM.pdf]

Supplementary information

Staphylococcus aureus lipoproteins promote abscess formation in mice, shielding bacteria from immune killing

Majd Mohammad[1*], Manli Na[1], Zhicheng Hu[1,2], Minh-Thu Nguyen[3,a], Pradeep Kumar Kopparapu[1], Anders Jarneborn[1,4], Anna Karlsson[1], Abukar Ali[1], Rille Pullerits[1,5], Friedrich Götz[3], Tao Jin[1,4]

[1] Department of Rheumatology and Inflammation Research, Institute of Medicine, Sahlgrenska Academy, University of Gothenburg, Gothenburg, Sweden

[2] Department of Microbiology and Immunology, The Affiliated Hospital of Guizhou Medical University, Guiyang, China

[3] Department of Microbial Genetics, Interfaculty Institute of Microbiology and Infection Medicine Tübingen (IMIT), University of Tübingen, Tübingen, Germany

[4] Department of Rheumatology, Sahlgrenska University Hospital, Gothenburg, Sweden

[5] Department of Clinical Immunology and Transfusion Medicine, Sahlgrenska University Hospital, Gothenburg, Sweden

[a] Current affiliation: Section of Medical and Geographical Infectiology, Institute of Medical Microbilogy, University Hospital of Münster, Münster, Germany

* Corresponding author:

M.M.: majd.mohammad@rheuma.gu.se; Department of Rheumatology and Inflammation Research, University of Gothenburg, Guldhedsgatan 10A, S-413 46 Gothenburg, Sweden. Tel: +46-31-3426475.

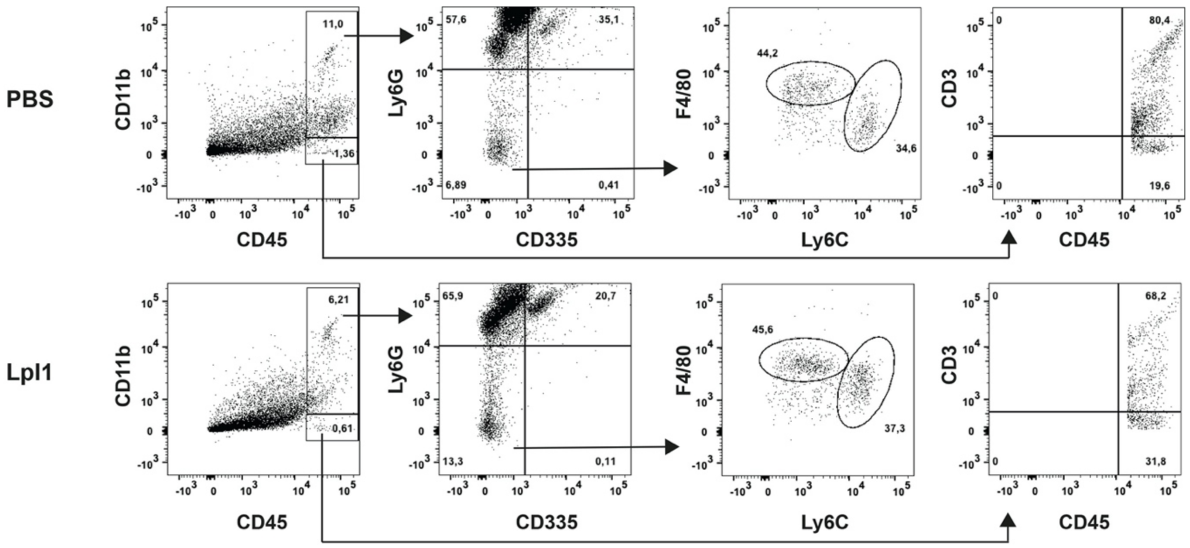

Supplementary Fig. 1: Migration of immune cells to the local skin following subcutaneous injection with *S. aureus* Lpp in TLR2 deficient mice as confirmed by flow cytometry analysis.

Representative FACS dot plots of cells isolated from auricular skin tissue following subcutaneous injection of phosphate-buffered saline (PBS) (upper panel) or purified *Staphylococcus aureus* lipoprotein, denoted as Lpl1 (1 µg) (lower panel) in the mouse ear in TLR2 deficient (TLR2$^{-/-}$) mice.

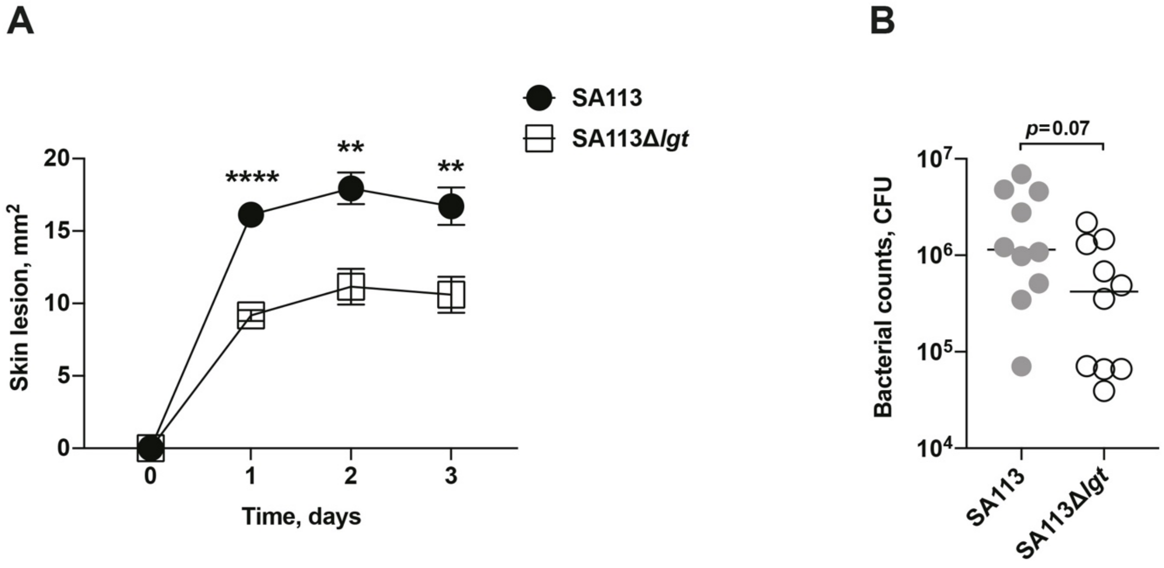

Supplementary Fig. 2: The reduced bacterial dose setting induces severe skin lesions in SA113 parental strain with a tendency of higher bacterial burden in local skin compared to its Δ*lgt* mutant strain.

(**A**) The skin lesion size (mm^2) in NMRI mice followed up to 3 days after subcutaneous (s.c.) skin injection with 50 µl of *Staphylococcus aureus* SA113 parental strain or SA113Δ*lgt* mutant strain ($7.5x10^6$ CFU/site; n = 10/group). (**B**) Bacterial counts in the supernatant of skin biopsy homogenates on day 3 after s.c. skin infection with SA113 parental strain or SA113Δ*lgt* mutant strain ($7.5x10^6$ CFU/site; n = 10/group) in NMRI mice. The data were pooled from 2 independent experiments. Statistical evaluations were performed using the Mann–Whitney U test, with data expressed as the mean ± standard error of the mean (A), or presented as scatterplot with line indicating median value (B). ***P* < 0.01; ****P* < 0.001.

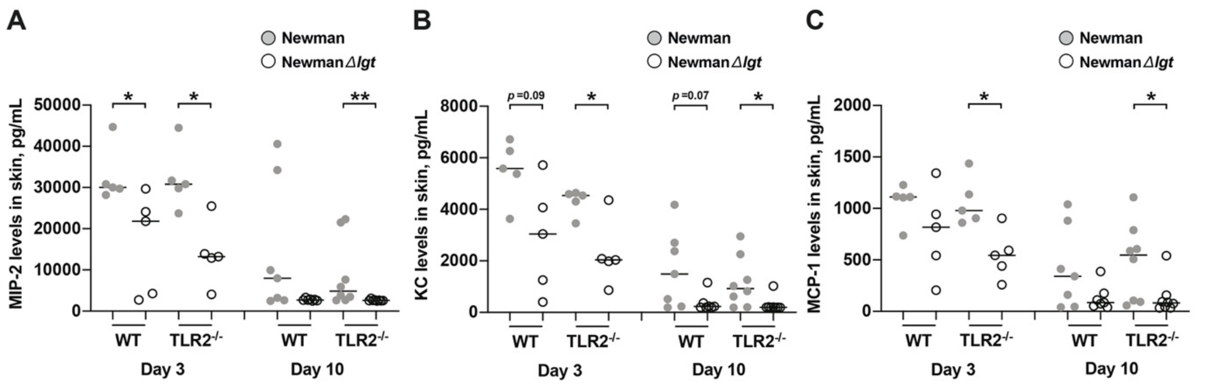

Supplementary Fig. 3: The levels of chemokines in skin tissues from mice infected with *Staphylococcus aureus* Newman or NewmanΔ*lgt* mutant strain.

The levels of (**A**) macrophage inflammatory protein-2 (MIP-2), (**B**) keratinocyte chemoattractant (KC) and (**C**) monocyte chemoattractant protein 1 (MCP-1) in the supernatant of skin biopsy homogenates of C57BL/6 wild-type (WT) mice and TLR2 deficient ($TLR2^{-/-}$) mice on day 3 (n = 5/group) and day 10 (n = 7-8/group) after subcutaneous (s.c.) skin injection with 20 µl of *Staphylococcus aureus* Newman parental strain or NewmanΔ*lgt* mutant strain (1.5 – 2.0 x 10^6 colony-forming units/site). The data were pooled from 2 independent experiments. Statistical evaluations were performed using the Mann–Whitney U test, with data presented as scatterplot with line indicating median value. *$P < 0.05$; **$P < 0.01$.

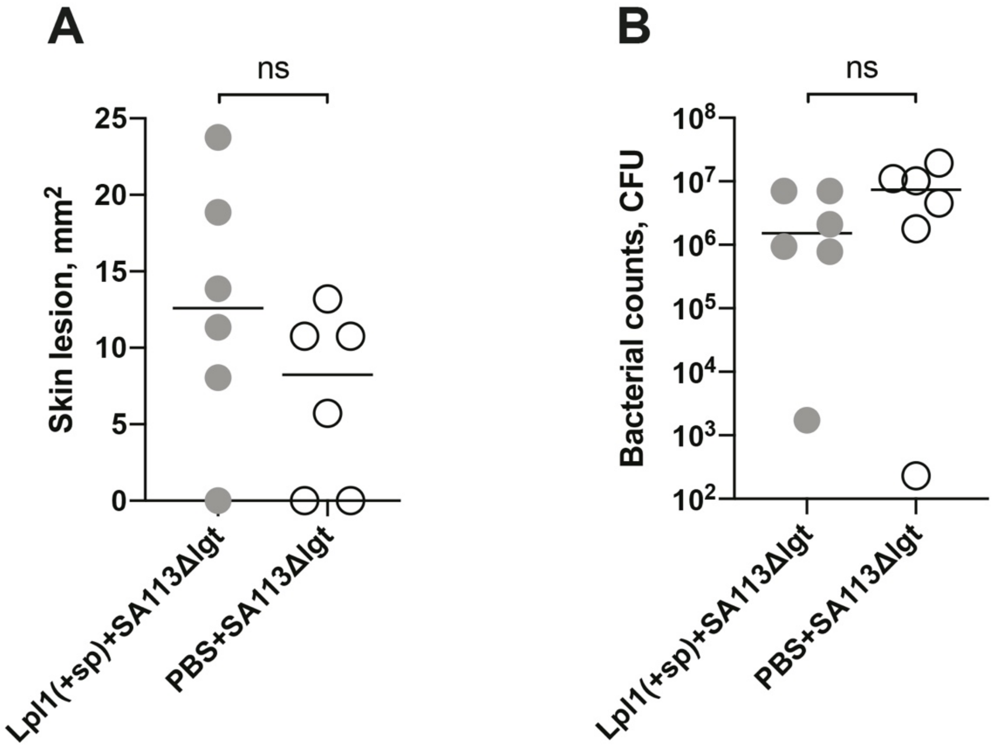

Supplementary Fig. 4: **Similar lesion severity and bacterial burden at the early disease course following coinjection of live *S. aureus* with either Lpp or control substance.**

(**A**) The skin lesion size (mm^2) and (**B**) bacterial counts in the supernatant of skin biopsy homogenates of NMRI mice (n = 6/group) 1 day after subcutaneous skin coinjection with 20 µl of *Staphylococcus aureus* (*S. aureus*) SA113Δ*lgt* mutant strain ($2.4 x 10^6$ colony-forming units [CFU/site]) with either phosphate-buffered saline (PBS) or purified *S. aureus* lipoprotein, denoted as Lpl1(+sp) (5 µg/site). Statistical evaluations were performed using the Mann–Whitney U test, with data presented as scatterplot with line indicating median value. ns = not significant.

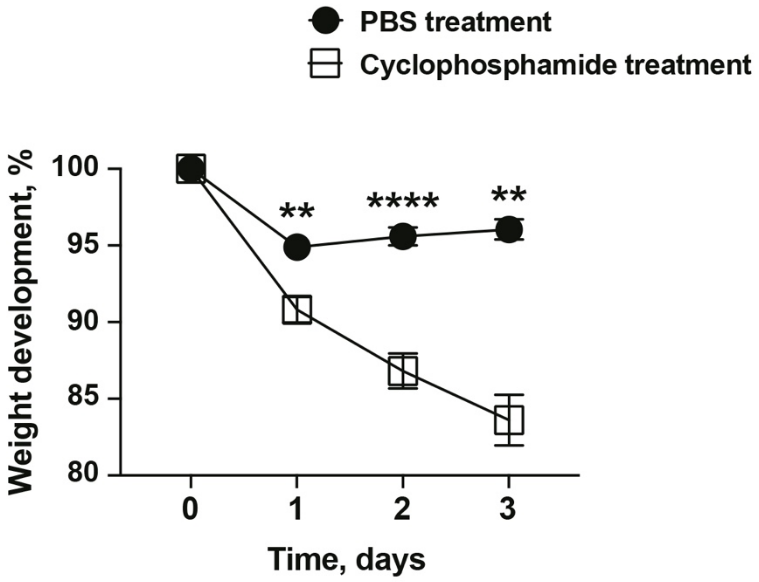

Supplementary Fig. 5: Leukocyte depletion induces severe weight loss in mice.

The weight changes up to 3 days after subcutaneous (s.c.) skin injection with 20 µl of *Staphylococcus aureus* Newman parental strain or NewmanΔ*lgt* mutant strain (2x10^6 colony-forming units (CFU)/site) in NMRI mice depleted of leukocytes using cyclophosphamide or treated with phosphate-buffered saline (PBS) as control (n = 8-10/group). The data were pooled from 2 independent experiments. Statistical evaluations were performed using the Mann–Whitney U test, with data expressed as the mean ± standard error of the mean. ***P* < 0.01; *****P* < 0.0001.

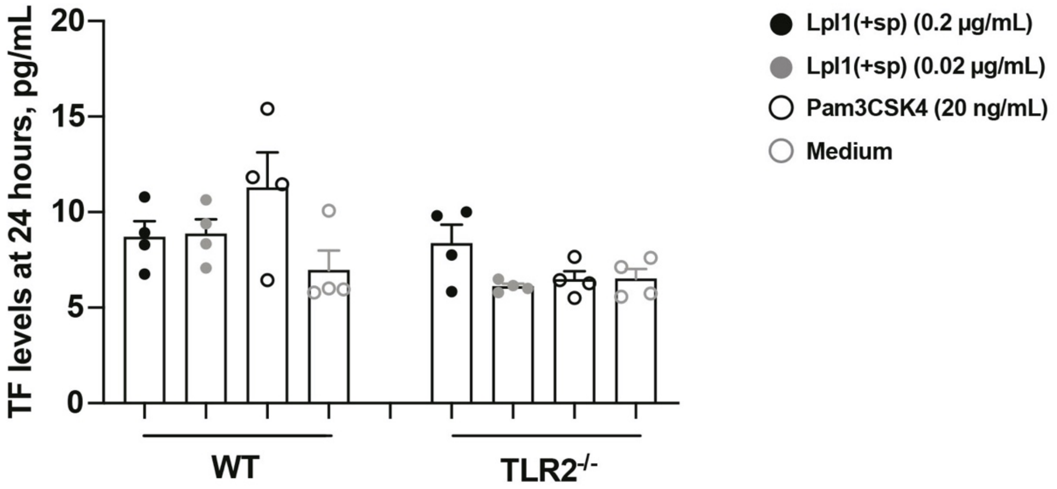

Supplementary Fig. 6: *Staphylococcus aureus* lipoproteins do not induce tissue factor expression in mouse peritoneal macrophages.

The levels of tissue factor (TF) in the supernatants collected from C57BL/6 wildtype (WT) and TLR2 deficient ($TLR2^{-/-}$) (n = 4/group) mouse peritoneal macrophage cell cultures ($5x10^5$ cells/mL) after stimulation for 24 hours with purified *Staphylococcus aureus* lipoprotein, denoted as Lpl1(+sp) (0.02 or 0.2 µg/mL), Pam3CSK4 (20 ng/mL), or culture medium. Statistical evaluations were performed using the Mann–Whitney U test, with data expressed as the mean ± standard error of the mean.

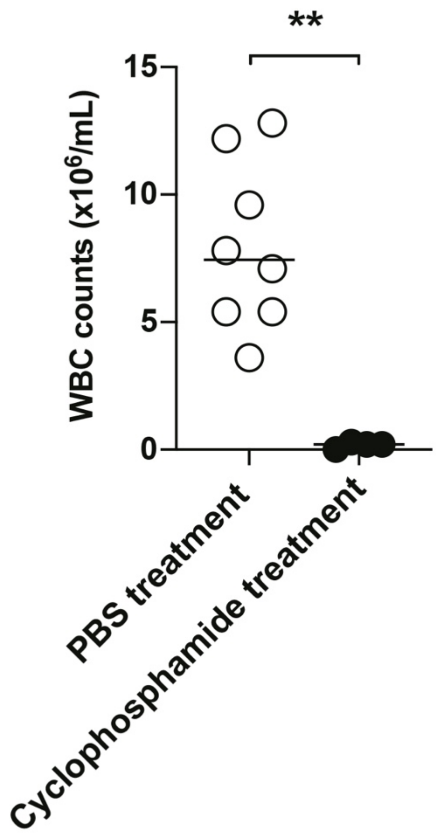

Supplementary Fig. 7: Efficacy of leukocyte depletion in mice by cyclophosphamide treatment.

NMRI mice were treated with either PBS as a control substance, or with cyclophosphamide in order to deplete leukocytes, four days and one day prior to the skin infection with *Staphylococcus aureus*. The mice were sacrificed on day 3 postinfection and the level of peripheral mouse blood leukocytes were thereafter assessed in a cell counter (n = 4-8/group). The data were pooled from 2 independent experiments. Statistical evaluations were performed using the Mann–Whitney U test, with data presented as scatterplot with line indicating median value (B). $^{**}P < 0.01$.

Supplementary Table 1: Incidence of bacterial counts from various organs on day 3 postinfection in fibrinogen-depleted mice (using Ancrod) or in PBS-treated controls (n = 4/group) after subcutaneous infection with *Staphylococcus aureus*.

	Blood	Kidneys	Liver	Spleen	Lungs
Ancrod	0/4	0/4	0/4	0/4	0/4
PBS	0/4	1/4	1/4	1/4	1/4
