# Supplementary material for: Staphylococcus aureus lipoproteins promote abscess formation in mice, shielding bacteria from immune killing
Source: Commun Biol. 2021 Mar 30;4:432. doi: 10.1038/s42003-021-01947-z (PMC8010101; doi:10.1038/s42003-021-01947-z)
Supplement: Supplementary file 3 — Description of Additional Supplementary Files [file 42003_2021_1947_MOESM3_ESM.pdf]

Description of Additional Supplementary Files

File Name: Supplementary Data 1

Description: **Sheet (1)** Raw data for figure 1a: skin lesion size. **Sheet (2)** Raw data for figure 1c: skin abscess frequency. **Sheet (3)** Raw data for figure 1e-g: ELISA data from murine skin tissue. **Sheet (4)** Raw data for figure 1h: skin lesion size. **Sheet (5)** Raw data for figure 1i-k: ELISA data.

File Name: Supplementary Data 2

Description: **Sheet (1)** Raw data for figure 2b-f: flow cytometry data.

File Name: Supplementary Data 3

Description: **Sheet (1)** Raw data for figure 3a: skin lesion size. **Sheet (2)** Raw data for figure 3b: skin wound healing. **Sheet (3)** Raw data for figure 3c: bacterial load. **Sheet (4)** Raw data for figure 3d-g: ELISA data. **Sheet (5)** Raw data for figure 3h: skin lesion size. **Sheet (6)** Raw data for figure 3i: bacterial load. **Sheet (7)** Raw data for figure 3j-m: ELISA data.

File Name: Supplementary Data 4

Description: **Sheet (1)** Raw data for figure 4a-b: skin lesion size. **Sheet (2)** Raw data for figure 4c-d: bacterial load.

File Name: Supplementary Data 5

Description: **Sheet (1)** Raw data for figure 5a: skin lesion size. **Sheet (2)** Raw data for figure 5b: skin abscess frequency. **Sheet (3)** Raw data for figure 5c: bacterial load. **Sheet (4)** Raw data for figure 5d-g: ELISA data. **Sheet (5)** Raw data for figure 5h: skin lesion size. **Sheet (6)** Raw data for figure 5i: bacterial load. **Sheet (7)** Raw data for figure 5j-m: ELISA data.

File Name: Supplementary Data 6

Description: **Sheet (1)** Raw data for figure 6a: skin lesion size in PBS- or cyclophosphamide treated NMRI mice. **Sheet (2)** Raw data for figure 6b: bacterial load in local skin tissues of PBS- or cyclophosphamide treated NMRI mice. Description: **Sheet (3)** Raw data for figure 6c: skin lesion size in Balb/c or SCID mice. **Sheet (4)** Raw data for figure 6d: bacterial load in local skin tissues of Balb/c or SCID mice.

File Name: Supplementary Data 7

Description: **Sheet (1)** Raw data for figure 7a-f: ELISA data from murine skin tissue. **Sheet (2)** Raw data for figure 7g: ELISA data from *in vitro* stimulation of murine peritoneal macrophages. **Sheet (3)** Raw data for figure 7h: skin lesion size in PBS- or Ancrod treated NMRI mice. **Sheet (4)** Raw data for figure 7i: bacterial load in local skin tissues of PBS- or Ancrod treated NMRI mice.
