# Supplementary material for: Staphylococcus aureus lipoproteins promote abscess formation in mice, shielding bacteria from immune killing
Source: Commun Biol. 2021 Mar 30;4:432. doi: 10.1038/s42003-021-01947-z (PMC8010101; doi:10.1038/s42003-021-01947-z)
Supplement: Supplementary file 11 — Reporting Summary [file 42003_2021_1947_MOESM11_ESM.pdf]

nature research

Corresponding author(s): Majd Mohammad, E-mail: majd.mohammad@rheuma.gu.se

Last updated by author(s): Feb 23, 2021

Reporting Summary

Nature Research wishes to improve the reproducibility of the work that we publish. This form provides structure for consistency and transparency in reporting. For further information on Nature Research policies, see our Editorial Policies and the Editorial Policy Checklist.

Statistics

For all statistical analyses, confirm that the following items are present in the figure legend, table legend, main text, or Methods section.

n/a	Confirmed	
☐	☒	The exact sample size (n) for each experimental group/condition, given as a discrete number and unit of measurement
☐	☒	A statement on whether measurements were taken from distinct samples or whether the same sample was measured repeatedly
☐	☒	The statistical test(s) used AND whether they are one- or two-sided<br>*Only common tests should be described solely by name; describe more complex techniques in the Methods section.*
☒	☐	A description of all covariates tested
☒	☐	A description of any assumptions or corrections, such as tests of normality and adjustment for multiple comparisons
☐	☒	A full description of the statistical parameters including central tendency (e.g. means) or other basic estimates (e.g. regression coefficient) AND variation (e.g. standard deviation) or associated estimates of uncertainty (e.g. confidence intervals)
☒	☐	For null hypothesis testing, the test statistic (e.g. F, t, r) with confidence intervals, effect sizes, degrees of freedom and P value noted<br>*Give P values as exact values whenever suitable.*
☒	☐	For Bayesian analysis, information on the choice of priors and Markov chain Monte Carlo settings
☒	☐	For hierarchical and complex designs, identification of the appropriate level for tests and full reporting of outcomes
☒	☐	Estimates of effect sizes (e.g. Cohen's d, Pearson's r), indicating how they were calculated

Our web collection on statistics for biologists contains articles on many of the points above.

Software and code

Policy information about availability of computer code

Data collection	N/A
Data analysis	N/A

For manuscripts utilizing custom algorithms or software that are central to the research but not yet described in published literature, software must be made available to editors and reviewers. We strongly encourage code deposition in a community repository (e.g. GitHub). See the Nature Research guidelines for submitting code & software for further information.

Data

Policy information about availability of data

All manuscripts must include a data availability statement. This statement should provide the following information, where applicable:

- Accession codes, unique identifiers, or web links for publicly available datasets
- A list of figures that have associated raw data
- A description of any restrictions on data availability

The authors declare that the main data supporting the findings of this study are available within the article and its Supplementary Information files. Extra data are available from the corresponding author upon request.

Field-specific reporting

Life sciences study design

All studies must disclose on these points even when the disclosure is negative.

Sample size	The sample sizes were chosen or calculated based on the results from previous experiments.
Data exclusions	No data was excluded.
Replication	The experiments were repeated at least two times. All attempts at replication were successful.
Randomization	Yes, animals in all experiments were randomized.
Blinding	All investigators were blinded to the treatment groups.

Reporting for specific materials, systems and methods

We require information from authors about some types of materials, experimental systems and methods used in many studies. Here, indicate whether each material, system or method listed is relevant to your study. If you are not sure if a list item applies to your research, read the appropriate section before selecting a response.

Materials & experimental systems

n/a	Involved in the study	
☐	☒	Antibodies
☒	☐	Eukaryotic cell lines
☒	☐	Palaeontology and archaeology
☐	☒	Animals and other organisms
☒	☐	Human research participants
☒	☐	Clinical data
☒	☐	Dual use research of concern

Methods

n/a	Involved in the study	
☒	☐	ChIP-seq
☐	☒	Flow cytometry
☒	☐	MRI-based neuroimaging

Antibodies

Antibodies used	The following antibodies were used: APC-R700-conjugated rat anti-mouse CD45 (565478; 30-F11; BD Biosciences), V450-conjugated rat anti-mouse CD11b (560455; Clone: M1/70; BD Biosciences), APC-conjugated rat anti-mouse F4/80 (123116; Clone: BM8; BioLegend), PE-Cy7-conjugated rat anti-mouse Ly-6G (560601; Clone: 1A8; BD Biosciences), BV605-conjugated rat anti-mouse Ly-6C (563011; Clone: AL-21; BD Biosciences), PerCP-Cy5.5-conjugated rat anti-mouse CD335 (560800; 29A1.4; BD Biosciences), and FITC-conjugated rat anti-mouse CD3 (561798; Clone: 17A2; BD Biosciences).
Validation	The different antibodies have each been validated previously. Please see below:<br>APC-R700 Rat Anti-Mouse CD45: Lagasse et al, 2000 (PMID: 11062533); V450 Rat anti-CD11b: Lagasse et al, 1996 (PMID: 8890901); APC anti-mouse F4/80: Kobayashi et al, 2008 (PMID: 18372338); PE-Cy7 Rat Anti-Mouse Ly-6G: Fleming et al, 1993 (PMID: 8360469); BV605 Rat Anti-Mouse Ly-6C: Tough et al, 1996 (PMID: 8658169); PerCP-Cy5.5 Rat Anti-Mouse CD335:Walzer et al, 2007 (PMID: 17360655); FITC Rat Anti-Mouse CD3: Mysliwietz et al, 1992 (PMID: 1358260). In addition, we have previously also validated some of the antibodies used in this manuscript: Mohammad et al, 2019 (PMID: 31226163).

Animals and other organisms

Policy information about studies involving animals; ARRIVE guidelines recommended for reporting animal research

Laboratory animals	Female NMRI mice, aged 6-10 weeks, were purchased from Envigo (Venray, Netherlands), gender- and age-matched 6- to 10-week-old C57BL/6 wild-type mice and Toll-like receptor 2-deficient B6.129-Tlr2tm1Kir/J (TLR2-/-) mice were purchased from Charles River Laboratories (Sulzfeld, Germany) and The Jackson laboratory (Bar Harbor, Maine, USA), respectively. Female CB17-SCID mice and Balb/c mice, aged 8 weeks, were purchased from Charles River Laboratories (Sulzfeld, Germany).
Wild animals	N/A
Field-collected samples	N/A
Ethics oversight	Mouse studies were reviewed and approved by the Ethics Committee of Animal Research of Gothenburg. Mouse experiments were conducted in accordance with recommendations listed in the Swedish Board of Agriculture's regulations and recommendations on animal experiments.

Note that full information on the approval of the study protocol must also be provided in the manuscript.

Flow Cytometry

Plots

Confirm that:

- [x] The axis labels state the marker and fluorochrome used (e.g. CD4-FITC).
- [x] The axis scales are clearly visible. Include numbers along axes only for bottom left plot of group (a 'group' is an analysis of identical markers).
- [x] All plots are contour plots with outliers or pseudocolor plots.
- [x] A numerical value for number of cells or percentage (with statistics) is provided.

Methodology

Sample preparation	A dose of 1 μg of Lpl1(+sp) in 10 μl of PBS or PBS alone as internal control, were s.c. injected into the auricle of anesthetized C57BL/6 wild-type (n = 6), and TLR2-/- (n = 5) mice. On day 1 after injection, the ear tissues were collected, placed in RPMI medium (Fisher Scientific), and subjected to enzymatic digestion with 4 mg/ml Collagenase IV (Fisher Scientific) and 0.4 mg/ml DNase I (Sigma-Aldrich) in RPMI medium, followed by incubation for 1 hour at 37°C with shaking. A single-cell suspension was obtained after the tissue was homogenized and passed through a 40 μm cell strainer (Becton Dickinson).
Instrument	Cells were acquired on a BD FACSLyric flow cytometer (BD Biosciences).
Software	Data was analyzed using FlowJo version 10.1 software (Tree Star, Ashland, USA).
Cell population abundance	N/A
Gating strategy	The gating strategy was provided in Figure 2 and Supplementary figure 1.

- [x] Tick this box to confirm that a figure exemplifying the gating strategy is provided in the Supplementary Information.
